# Personal, Normative, and Access-Related Correlates of Fruit and Vegetable Purchase Intention in Peruvian Adults

**DOI:** 10.3390/nu18183033

**Published:** 2026-09-16

**Authors:** Eliana Contreras-López, Rafael Orlando Florián-Castro, José Alberto Nuñez Ramos, Ana Veliz Barandiaran, Jacksaint Saintila

**Affiliations:** 1 Facultad de Farmacia y Bioquímica, Universidad Nacional Mayor de San Marcos, Lima 15081, Peru; econtrerasl@unmsm.edu.pe; 2 Escuela de Posgrado, Universidad San Ignacio de Loyola, Lima 15024, Peru; rafael.florian@usil.pe; 3 Intihuatana Pharmaceutical SAC, Av. José Pardo 640 Dpto 801, Miraflores, Lima 15074, Peru; anunez@intipha.com; 4 Servicio de Nutrición, British American Hospital, Lima 15073, Peru; aveliz@angloamericana.com.pe; 5 Maestría en Nutrición Humana, Escuela de Posgrado, Universidad Peruana Unión, Lima 15464, Peru

**Keywords:** purchase intention, fruit, vegetables, health consciousness, perceived behavioral control, theory of planned behavior, Peru

## Abstract

**Background/Objectives:** Fruit and vegetable purchase intention may reflect personal, normative, and access-related factors, yet evidence from Peru remains limited. This study aimed to examine associations of health consciousness, family influence, perceived behavioral control, and perceived convenience and availability with attitude and fruit and vegetable purchase intention, including indirect statistical associations through attitude. **Methods:** This cross-sectional study used non-probability convenience sampling to recruit 458 adults residing in Peru; 73.8% were female, 76.9% had university education, and 66.2% resided in Lima. Standardized composite scores were analyzed in a path model adjusted for sex, age group, education, marital status, household income, and region. HC3 robust standard errors and 5000 bootstrap resamples were used. **Results:** Health consciousness (β = 0.331), perceived behavioral control (β = 0.228), and perceived convenience and availability (β = 0.096) were positively associated with attitude, whereas family influence was not. Attitude was positively associated with purchase intention (β = 0.568), while direct associations of the four antecedents with purchase intention were not supported. Positive indirect statistical associations through attitude were observed for health consciousness (β = 0.188, 95% CI: 0.117–0.265), perceived behavioral control (β = 0.130, 95% CI: 0.073–0.190), and perceived convenience and availability (β = 0.055, 95% CI: 0.010–0.104). The model explained 29.4% of attitude and 43.2% of purchase-intention variance. **Conclusions:** Health consciousness and perceived behavioral control showed the most consistent associations. Given the non-random and demographically imbalanced sample, findings should not be generalized to all Peruvian adults or interpreted as causal pathways or actual purchasing behavior.

## 1. Introduction

A healthy diet is a fundamental component of preventing noncommunicable diseases and reducing the risk of premature mortality. Within healthy dietary patterns, fruits and vegetables provide dietary fiber, vitamins, minerals, and a wide range of bioactive compounds that support metabolic and cardiovascular health [1]. The World Health Organization recommends at least 400 g of fruits and vegetables per day for individuals aged 10 years and older [2], and prospective evidence links higher intake with lower all-cause and cardiovascular mortality [3]. In Peru, however, the 2024 Demographic and Family Health Survey (ENDES) reported that only 8.9% of people aged 15 years and older consumed at least five daily servings of fruits and vegetables, with lower prevalence in rural than urban areas [4].

Low fruit and vegetable intake reflects more than nutritional knowledge alone; food-related decisions are shaped by interacting personal, social, and contextual influences [5,6]. Purchase intention is relevant in this decision process because it represents a person’s willingness or readiness to acquire a product in the foreseeable future [7,8,9]. Intention is not equivalent to actual purchasing or dietary intake, and intention–behavior gaps are well documented [10,11]. Nevertheless, studying purchase intention can identify correlates of consumers’ predisposition to choose fruits and vegetables at a stage that precedes, but does not guarantee, purchasing behavior [10,11,12].

The Theory of Planned Behavior (TPB) provides a useful reference framework for understanding behavioral intention. In the canonical TPB, attitude, subjective norm, and perceived behavioral control are parallel antecedents of intention [9,13]. Meta-analytic evidence supports the relevance of these constructs for dietary intentions and food-related behaviors, although their relative importance varies across populations and behaviors [14,15]. The present study was informed by this framework but was not intended as a formal extension or complete test of the TPB. Instead, it jointly examined selected personal, family-related normative, control-related, and perceived access-related factors that may be relevant to fruit and vegetable purchase intention.

Previous studies provide support for examining these domains together. Health consciousness has been associated with favorable attitudes and stronger intentions toward healthy or organic foods [16,17,18,19,20]. Attitude has frequently shown a positive association with purchase intention, including for organic vegetables in Brazil and domestically produced fruits and vegetables in Portugal [21,22,23]. Perceived behavioral control has also been associated with dietary and food-purchasing intentions [14,15,17,18,23,24,25]. However, evidence specifically concerning conventional fruit and vegetable purchase intention remains limited, and much of the available literature focuses on organic foods, younger consumers, or populations outside Latin America [18,21,22,23,26,27]. Peruvian research has more often focused on inadequate fruit and vegetable intake or broader healthy-food purchase intention rather than on the purchase intention for fruits and vegetables specifically [17,28,29].

The constructs were delimited to address important conceptual distinctions. Family influence captured approval, expectations, recommendations, and perceived influence from a partner or family and was therefore treated as a family-specific normative influence related to, but not equivalent to, the conventional TPB subjective-norm construct [30,31,32,33,34]. Perceived behavioral control was operationalized primarily as personal control and confidence in the ability to purchase fruits and vegetables. In contrast, perceived convenience and availability captured perceived external retail conditions, including proximity of stores, product variety, and perceived availability [34,35,36,37,38,39,40]. These are subjective perceptions of the shopping environment and should not be interpreted as objective measures of retail access. Because associations of these antecedent factors with attitude are not all specified by the canonical TPB, the paths to attitude were treated as additional theoretically informed associations rather than as standard TPB relations.

The study therefore focused on the joint examination of these personal, family-related normative, perceived-control, and perceived-access correlates in adults residing in Peru. The primary aim was to examine their associations with attitude and fruit and vegetable purchase intention in a sociodemographically adjusted cross-sectional path model. A secondary aim was to quantify model-based indirect statistical associations through attitude while explicitly treating them as cross-sectional associations rather than evidence of temporal or causal mediation.

## 2. Materials and Methods

### 2.1. Study Design, Setting, and Participants

A quantitative, observational, cross-sectional study was conducted among adults residing in Peru. Data were collected through a self-administered online questionnaire developed in QuestionPro (web-based platform; QuestionPro Inc., Austin, TX, USA). The survey link was disseminated through WhatsApp groups and Facebook Messenger; accordingly, recruitment depended on voluntary participation and access to the electronic survey. The source population included adults from Lima, Callao, and other regions of Peru who could access the survey link.

Eligible participants were individuals aged 18 years or older who resided in Peru, were able to understand the questionnaire in Spanish, and provided electronic informed consent. Questionnaires without consent, incomplete responses, and records from individuals who did not meet the eligibility criteria were excluded. The questionnaire collected the six psychosocial constructs together with age group, sex, educational level, marital status, employment status, monthly household income, region of residence, and self-reported physical activity level.

### 2.2. Sample Size and Sampling Procedure

A non-probability convenience sampling approach was used. The minimum sample size required for the initially specified six-factor measurement model was estimated a priori using the *semPower* package in R and an RMSEA-based approach. The calculation assumed an RMSEA of 0.05, a significance level of 0.05, statistical power of 0.80, and 174 degrees of freedom, corresponding to the original model with six constructs and 21 indicators. Under these assumptions, the minimum required sample was 121 participants. The final analytical sample of 458 adults exceeded this minimum.

Before the main data collection, the preliminary questionnaire was pilot-tested among 30 adults who met the main eligibility criteria but were not included in the final analytical sample. The pilot test assessed the overall comprehensibility of the items and instructions, questionnaire sequence, functionality of the electronic form, and completion time. Based on participant feedback, only minor wording and formatting adjustments were made to improve clarity; no dimensions were added or removed, and the conceptual meaning of the items was not modified. The pilot test was not intended to evaluate the psychometric properties of the scales.

### 2.3. Psychosocial Measures

The questionnaire contained 21 items distributed across six constructs: health consciousness (HC), family influence (FI), perceived behavioral control (PBC), perceived convenience and availability (PCA), attitude (ATT), and purchase intention (PI). Items were drawn from previously used instruments and adapted to the context of purchasing fruits and vegetables. Responses used a five-point Likert-type scale from 1 = strongly disagree to 5 = strongly agree. Negatively worded items were reverse-coded so that higher values consistently represented higher levels of the corresponding construct. Primary composite scores were calculated as the mean of the items retained for the primary structural analysis. Complete wording, English translations, coding, analytical status, and sources are reported in Supplementary Table S1, Questionnaire items, English translations, coding, analytical status, and sources.

#### 2.3.1. Health Consciousness

HC was initially assessed using three items adapted from Yadav and Pathak [18]. HC2 was negatively worded and reverse-coded. In the reproduced ordinal measurement assessment, HC2 showed comparatively weak convergence with the other HC indicators; therefore, the primary HC composite used HC1 and HC3. The two retained items showed an ordinal Spearman–Brown coefficient of 0.731. HC2 remained in the complete 21-item measurement model and in sensitivity analyses, so its exclusion from the primary composite was not used as a criterion for selecting favorable structural results.

#### 2.3.2. Family Influence

FI was assessed using four items adopted from Chauke and Duh [32]. The items captured approval, expectations, influence, and recommendations from a partner or family regarding fruit and vegetable purchase or consumption. All four items were retained in the primary composite. Ordinal McDonald’s total omega was 0.744. Because these items refer specifically to family referents and combine several forms of interpersonal influence, FI was interpreted as a family-specific normative construct rather than as a direct operationalization of the full TPB subjective norm.

#### 2.3.3. Perceived Behavioral Control

PBC was initially assessed using three items adapted from Yadav and Pathak [18]. PBC1 and PBC2 assessed personal control and confidence in purchasing fruits and vegetables. PBC3 was negatively worded and referred to insufficient resources and time; after reverse-coding, it showed poor coherence with the two personal-control indicators and was conceptually closer to external constraints. The primary composite therefore used PBC1 and PBC2, with an ordinal Spearman–Brown coefficient of 0.619. Because this reliability was borderline, findings involving PBC were interpreted cautiously and evaluated across multiple measurement specifications.

#### 2.3.4. Perceived Convenience and Availability

PCA was assessed using three items adapted from Kamboj et al. [27]. The items referred to proximity of retail outlets, variety of fruits and vegetables at usual shopping locations, and availability across stores or markets. All were worded as barriers and were reverse-coded, so higher scores indicated greater perceived convenience and availability. Ordinal McDonald’s total omega was 0.858. PCA represents perceived shopping-environment conditions and not objectively measured retail access.

#### 2.3.5. Attitude Toward Purchasing

ATT was assessed using four items adopted from Eberle et al. [21]. The items evaluated liking, favorable appraisal, and the perception that purchasing fruits and vegetables was a good or pleasant choice. All four items were retained, and ordinal McDonald’s total omega was 0.929.

#### 2.3.6. Purchase Intention

PI was initially assessed using four items adapted from Kamboj et al. [27]. PI1–PI3 assessed the respondent’s willingness, intended effort in the near future, and intention to continue purchasing fruits and vegetables. PI4 assessed recommendation of fruit and vegetable purchasing to another person and was excluded from the primary composite on content-validity grounds because it did not directly assess the respondent’s own purchase intention. PI2 was retained despite a comparatively low factor loading because it captured intended effort and a near-future time frame; its exclusion was examined separately in sensitivity analyses. The PI1–PI3 composite showed ordinal McDonald’s total omega of 0.799.

### 2.4. Background Variables and Covariates

Age was recorded in four categories (18–30, 31–40, 41–50, and 51–60 years). Sex, education, marital status, employment status, monthly household income, region of residence, and physical activity level were self-reported using the categories shown in Table 1. For adjusted analyses, education was recoded as secondary or less, technical, and university; marital status as single, with partner (cohabiting or married), and previously united (divorced or widowed); household income retained its six categories; and region was classified as Lima, Callao, or other regions. Sex and age group were retained as collected.

Physical activity was assessed using a single self-reported question: “How many minutes of physical activity do you perform in a day? (If you do not engage in physical activity every day, average the weekly activity you perform into a daily value)”. Examples of moderate physical activity included brisk walking, gardening, active household tasks, and general construction work, whereas examples of vigorous activity included running or jogging, gym-based activities, fast cycling, and competitive sports. Response options were: (1) no physical activity; (2) <30 min/day of moderate activity or <15 min/day of vigorous activity; and (3) ≥30 min/day of moderate activity or ≥15 min/day of vigorous activity. This single-item variable was used only for descriptive characterization and was not included in the primary adjustment set.

Employment status was also described but was not included in the primary adjustment because socioeconomic background was already represented by education and household income. The primary adjusted model therefore included sex, age group, education, marital status, household income, and region as background covariates rather than as TPB constructs.

### 2.5. Conceptual Model and Hypotheses

**Figure 1:**
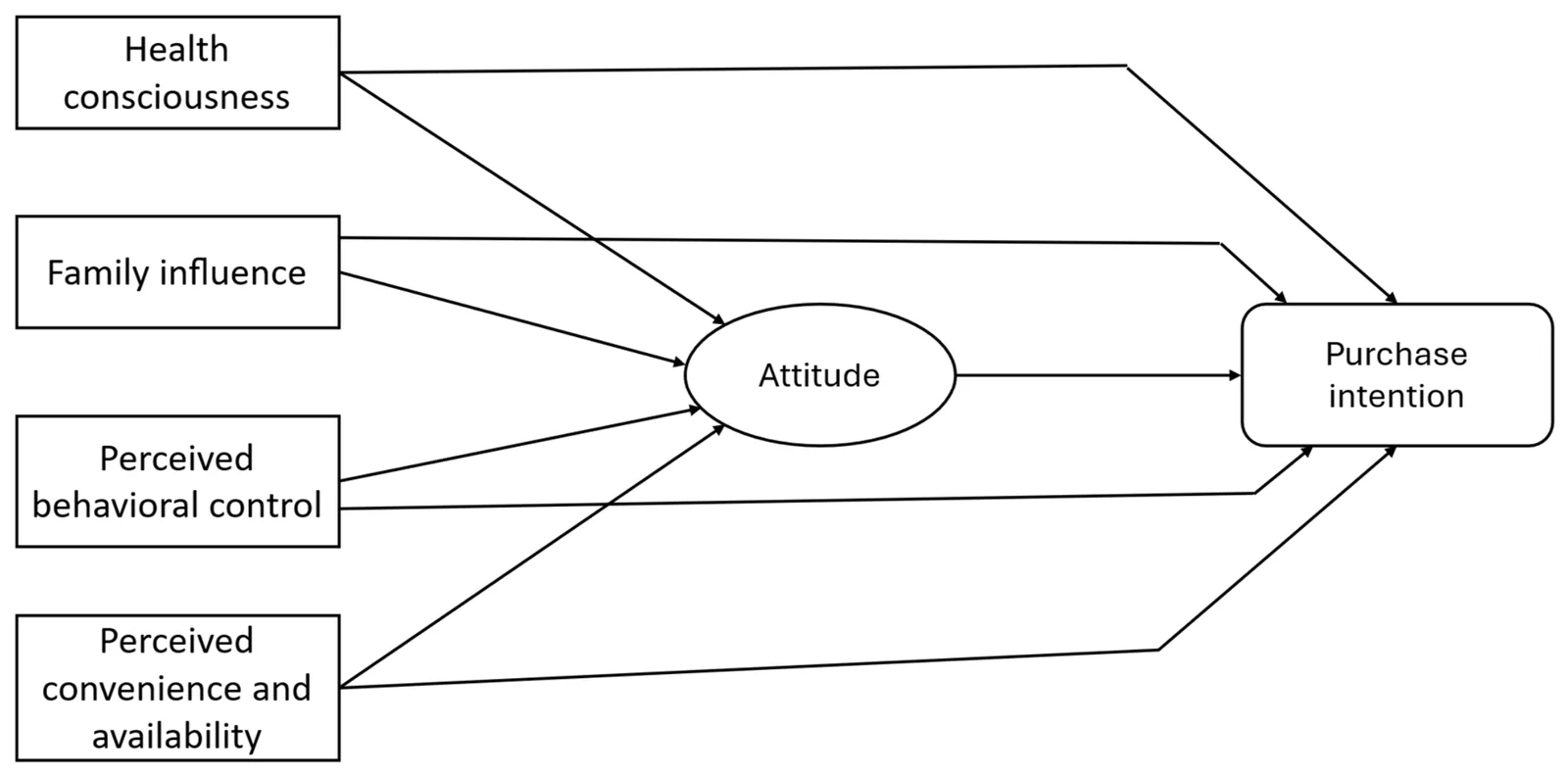
Conceptual model of the hypothesized direct and indirect statistical associations with fruit and vegetable purchase intention. Note. H1 concerns the associations of health consciousness, family influence, perceived behavioral control, and perceived convenience and availability with attitude. H2 concerns the associations of attitude and the four antecedent constructs with purchase intention. H3 concerns the indirect statistical associations of the four antecedent constructs with purchase intention through attitude.

The conceptual model treated ATT as the most proximal correlate of PI while also estimating direct associations of HC, FI, PBC, and PCA with both ATT and PI. The paths from the antecedent constructs to ATT were considered additional theoretically informed associations rather than canonical TPB paths. Three grouped hypotheses were evaluated: H1, HC, FI, PBC, and PCA are positively associated with ATT toward purchasing fruits and vegetables; H2, ATT and the four antecedent constructs are positively associated with PI; and H3, HC, FI, PBC, and PCA show positive indirect statistical associations with PI through ATT. Because the data were cross-sectional, H3 concerns model-based statistical associations and not temporal or causal mediation (Figure 1).

### 2.6. Ethical Considerations

The study protocol was reviewed and approved by the Ethics Committee of Universidad Peruana Union (Approval No. 2026-CE-EPG-00091; approved on 16 June 2026). The study was conducted in accordance with the ethical principles for research involving human participants and the Declaration of Helsinki. All participants provided informed consent before completing the questionnaire, and participation was voluntary and anonymous.

### 2.7. Statistical Analysis

Statistical analyses were performed in R version 4.4.1. Categorical background variables were summarized as absolute frequencies and relative frequencies (%). Primary construct scores were summarized using means, standard deviations, medians, interquartile ranges, and observed ranges. No missing values were identified in the 21 psychosocial items. Bivariate associations among the primary construct scores were described using Spearman rank correlations ($r_{s}$). Reliability was estimated using ordinal McDonald’s total omega for multi-item scores and the ordinal Spearman–Brown coefficient for two-item scores, based on polychoric correlations.

Measurement assessment began with the original 21-item six-factor confirmatory factor analysis (CFA), estimated for ordinal indicators using WLSMV with delta parameterization. Scaled chi-square, CFI, TLI, RMSEA with 95% confidence interval, and SRMR were reported together with convergence and admissibility diagnostics. Standardized loadings, composite reliability, AVE, latent-factor correlations, HTMT ratios based on polychoric correlations, and the Fornell–Larcker criterion were examined. Because ATT and PI showed substantial overlap, a focused two-factor ATT–PI model was compared with a one-factor specification using the robust WLSMV chi-square difference test. A hybrid reliability-corrected measurement model was additionally estimated as a sensitivity analysis: HC and PBC were represented by standardized two-item composites with residual error fixed according to their ordinal Spearman–Brown reliability, whereas FI, PCA, ATT, and PI were retained at the item level, with PI represented by PI1–PI3. Common-method variance was explored using a single-factor CFA and Harman’s unrotated single-factor solution based on the polychoric correlation matrix; these diagnostics were treated as evidence about single-factor dominance and not as proof that common-method variance was absent.

The primary structural analysis used standardized composite scores and two ordinary least-squares equations: ATT was regressed on HC, FI, PBC, PCA, and the background covariates, and PI was regressed on ATT, HC, FI, PBC, PCA, and the same covariates. The primary model was adjusted for sex, age group, education, marital status, household income, and region. Direct associations are reported as standardized coefficients (β) with HC3 heteroskedasticity-consistent standard errors, t statistics, p values, and 95% confidence intervals. Indirect and total statistical associations were estimated using 5000 nonparametric bootstrap resamples with percentile 95% confidence intervals; construct scores were re-standardized within each bootstrap resample. Multicollinearity was assessed using tolerance and VIF for focal one-degree-of-freedom predictors and adjusted GVIF for multi-category covariates.

Sensitivity analyses were conducted to evaluate the robustness of the findings across alternative analytical and measurement specifications rather than to identify the specification producing the largest coefficients or explained variance. These analyses included the corresponding unadjusted primary composite model; a full 21-item latent-variable SEM; the hybrid reliability-corrected latent SEM; and five adjusted composite specifications comprising the primary item set, the complete 21-item set, retention of PI4, exclusion of PI2, and a reduced-item specification excluding FI1, FI3, PBC1, and PI2, which had been flagged as potentially problematic indicators in an earlier measurement specification. Because this reduced-item specification left PBC represented by the single item PBC2, it was interpreted strictly as a sensitivity analysis and not as an alternative preferred measurement model. A geographic sensitivity analysis additionally combined Lima and Callao and compared this classification with other regions. Global fit indices from the structural SEMs were not interpreted as validation of individual structural paths because the construct-level structural relations essentially saturated the covariance structure. All tests were two-sided, with p < 0.05 used as a descriptive threshold for statistical support.

## 3. Results

A total of 458 participants were included. Most were female (73.8%) and university educated (76.9%), and 63.8% were aged 41–60 years. Married participants accounted for 41.0% of the sample, while 38.4% were single. Most participants were employees (65.5%), and 66.2% resided in Lima. Regarding physical activity, 42.4% reported <30 min/day of moderate activity or <15 min/day of vigorous activity, whereas 38.9% reported no physical activity (Table 1).

Table 2 summarizes the distributions of the primary study constructs. Attitude showed the highest mean score (M = 4.35, SD = 0.61), followed by purchase intention (M = 4.20, SD = 0.61), health consciousness (M = 4.05, SD = 0.79), and perceived behavioral control (M = 4.00, SD = 0.74). Family influence had a mean of 3.55 (SD = 0.74), whereas perceived convenience and availability showed the lowest mean (M = 3.47, SD = 0.97) and the greatest variability. The distributions of attitude and purchase intention were concentrated toward the upper end of the response scale.

Spearman correlation analyses showed that PI was positively correlated with HC ($r_{s}$ = 0.367, *p* < 0.001), FI ($r_{s}$ = 0.132, *p* = 0.005), PBC ($r_{s}$ = 0.222, *p* < 0.001), and ATT ($r_{s}$ = 0.572, *p* < 0.001), whereas its correlation with PCA was not statistically significant ($r_{s}$ = 0.044, *p* = 0.350). ATT was positively correlated with HC ($r_{s}$ = 0.502), PBC ($r_{s}$ = 0.321), and PCA ($r_{s}$ = 0.182; all *p* < 0.001). Primary-score reliability ranged from 0.619 to 0.929, with PBC showing the lowest reliability. Multicollinearity was not evident among the focal predictors in the adjusted PI model (tolerance = 0.706–0.884; VIF = 1.131–1.417); adjusted GVIF^(1/[2df]) values for multi-category covariates ranged from 1.059 to 1.085 (Table 3).

### 3.1. Measurement Model (CFA)

The complete wording, coding, and analytical status of the 21 questionnaire items are presented in Supplementary Table S1. The original 21-item six-factor CFA converged to an admissible solution, although the global fit indices provided mixed evidence of model fit: scaled χ^2^(174) = 952.12, *p* < 0.001, CFI = 0.940, TLI = 0.928, RMSEA = 0.099 (95% CI: 0.093–0.105), and SRMR = 0.083. As a complementary measurement-error sensitivity analysis, a hybrid reliability-corrected six-factor measurement model was also estimated; this specification showed somewhat higher CFI and TLI and a lower SRMR, while RMSEA remained elevated (scaled χ^2^(91) = 484.36, *p* < 0.001, CFI = 0.956, TLI = 0.942, RMSEA = 0.097, 95% CI: 0.089–0.106, SRMR = 0.073) (Supplementary Table S2).

In the original 21-item CFA, standardized factor loadings ranged from 0.359 to 0.915. Most indicators showed moderate-to-high loadings; however, HC2 (λ = 0.398), PBC1 (λ = 0.359), and PI2 (λ = 0.381) were comparatively weak. Construct-level measurement quality was heterogeneous: composite reliability ranged from 0.600 to 0.930 and AVE from 0.356 to 0.770, with HC, FI, and particularly PBC showing lower convergent validity. Reliability of the primary composite scores ranged from 0.619 for PBC to 0.929 for ATT (Supplementary Table S3).

Discriminant-validity diagnostics identified a particularly strong empirical overlap between attitude and purchase intention. Their latent correlation was 0.933 and the HTMT ratio was 0.925; moreover, the latent correlation exceeded the square roots of AVE for both ATT (0.877) and PI (0.807), indicating failure of the Fornell–Larcker criterion. Nevertheless, a focused two-factor ATT–PI model fit significantly better than a one-factor model in which all ATT and PI indicators were specified as a single construct, Δχ^2^(1) = 63.01, *p* < 0.001. Thus, ATT and PI were statistically distinguishable, but their discriminant validity remained limited. Detailed latent correlations, HTMT ratios, Fornell–Larcker results, and the focused ATT–PI model comparison are presented in Supplementary Table S4.

Common-method variance was additionally examined using complementary single-factor diagnostics. A single-factor CFA showed substantially poorer fit than the six-factor model (scaled χ^2^(189) = 3188.79, CFI = 0.769, TLI = 0.743, RMSEA = 0.186, SRMR = 0.136), and the robust model comparison favored the six-factor specification, Δχ^2^(15) = 1195.3, *p* < 0.001. In addition, an unrotated single factor based on the polychoric correlation matrix accounted for 33.1% of the total variance. These findings do not support an explanation in which a single general factor accounts for the observed covariance structure; however, common-method variance cannot be excluded because all constructs were self-reported in the same survey (Supplementary Table S8).

### 3.2. Adjusted Structural Path Model

**Figure 2:**
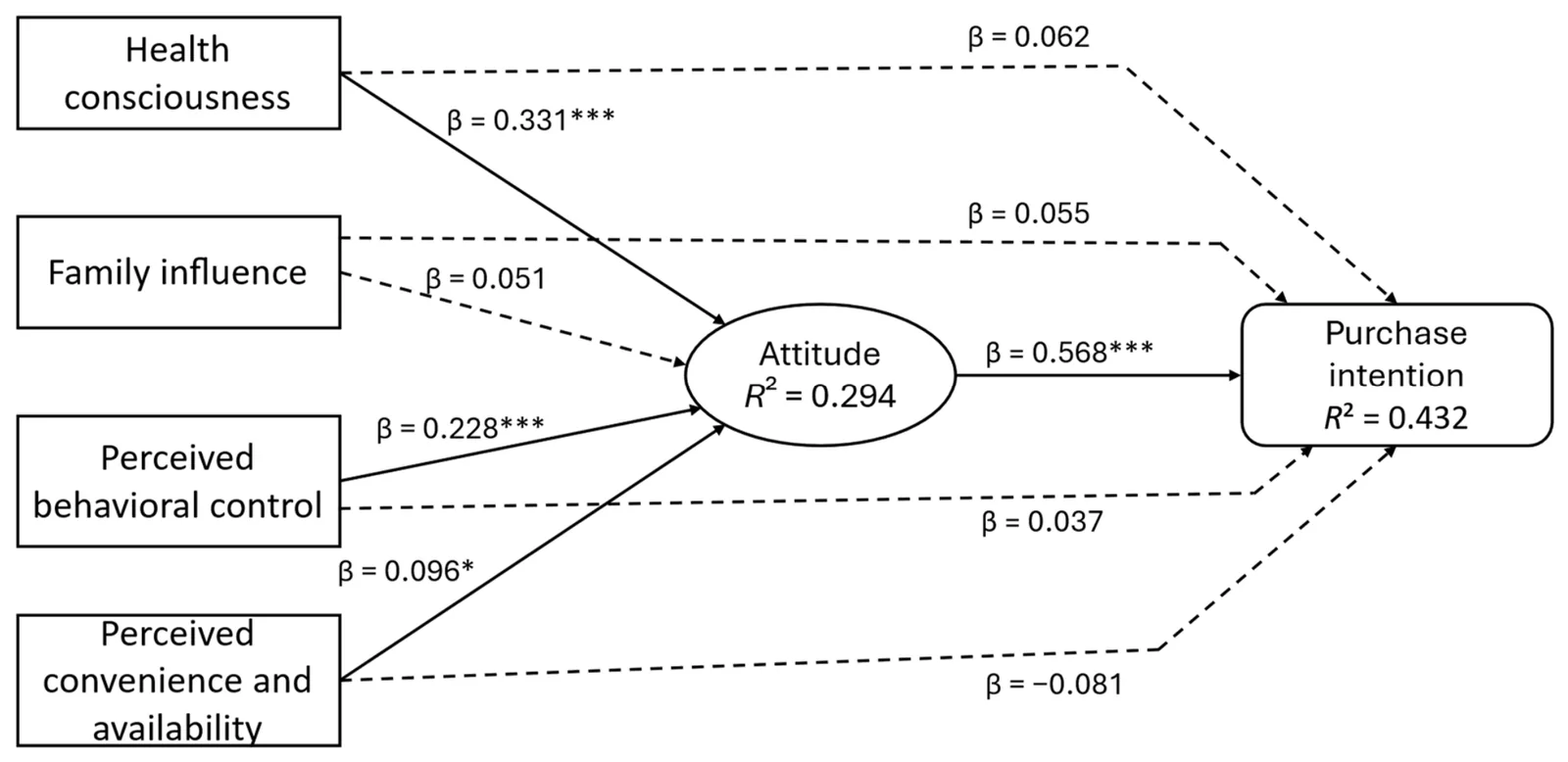
Sociodemographically adjusted path model of associations with fruit and vegetable purchase intention. Note. Standardized regression coefficients (β) are shown for the focal structural paths. Solid arrows indicate statistically significant associations (*p* < 0.05), whereas dashed arrows indicate associations not statistically supported. The model was adjusted for sex, age group, educational level, marital status, household-income category, and region of residence. Background covariates are not displayed for visual clarity. *R*^2^ = 0.294 for attitude and *R*^2^ = 0.432 for purchase intention. HC = health consciousness; FI = family influence; PBC = perceived behavioral control; PCA = perceived convenience and availability; ATT = attitude; PI = purchase intention. * *p* < 0.05; *** *p* < 0.001.

The primary structural analysis was the sociodemographically adjusted composite path model. The model explained 29.4% of the variance in attitude (*R*^2^ = 0.294) and 43.2% of the variance in purchase intention (*R*^2^ = 0.432). Health consciousness (β = 0.331, 95% CI: 0.197–0.465, *p* < 0.001), perceived behavioral control (β = 0.228, 95% CI: 0.120–0.337, *p* < 0.001), and perceived convenience and availability (β = 0.096, 95% CI: 0.014–0.178, *p* = 0.022) were positively associated with attitude, whereas family influence was not (β = 0.051, 95% CI: −0.054–0.155, *p* = 0.339). Attitude showed a positive association with purchase intention (β = 0.568, 95% CI: 0.463–0.674, *p* < 0.001). In contrast, the direct associations of health consciousness (β = 0.062, *p* = 0.265), family influence (β = 0.055, *p* = 0.210), perceived behavioral control (β = 0.037, *p* = 0.429), and perceived convenience and availability (β = −0.081, *p* = 0.053) with purchase intention were not statistically supported (Table 4 and Figure 2).

Positive indirect statistical associations with purchase intention through attitude were observed for health consciousness (β = 0.188, bootstrap 95% CI: 0.117–0.265), perceived behavioral control (β = 0.130, 95% CI: 0.073–0.190), and perceived convenience and availability (β = 0.055, 95% CI: 0.010–0.104), whereas the corresponding indirect association for family influence was not supported (β = 0.029, 95% CI: −0.025–0.082). Total associations with purchase intention were positive for health consciousness (β = 0.250, 95% CI: 0.141–0.361) and perceived behavioral control (β = 0.167, 95% CI: 0.056–0.266), whereas the total associations for family influence (β = 0.084, 95% CI: −0.020–0.186) and perceived convenience and availability (β = −0.026, 95% CI: −0.119–0.065) were not statistically supported (Table 4).

The corresponding unadjusted composite path model yielded a closely similar pattern. It explained 24.7% of the variance in ATT and 39.6% of the variance in PI, and positive indirect statistical associations through ATT were again observed for HC, PBC, and PCA. The total association for FI was small and positive in the unadjusted model but was not retained after sociodemographic adjustment, indicating limited robustness (Supplementary Table S5).

Latent-variable SEM sensitivity analyses further supported the principal findings for HC and PBC. In the full 21-item latent SEM, HC (β = 0.433, *p* < 0.001) and PBC (β = 0.445, *p* < 0.001) were positively associated with ATT, whereas FI and PCA were not. In the hybrid reliability-corrected latent SEM, HC (β = 0.361, *p* < 0.001), PBC (β = 0.332, *p* < 0.001), and PCA (β = 0.186, *p* < 0.001) were positively associated with ATT. ATT showed very large positive associations with PI in both latent models (β = 0.881 and 0.931, respectively). Positive indirect statistical associations through ATT were consistently observed for HC and PBC, whereas findings involving PCA and the direct PBC–PI path varied across measurement specifications (Supplementary Table S6). The magnitude of the ATT–PI coefficients in these latent models should be interpreted cautiously in view of the limited ATT–PI discriminant validity documented in Supplementary Table S4.

Finally, sensitivity analyses using alternative item specifications supported the stability of the positive associations of HC and PBC with ATT and of the corresponding indirect statistical associations with PI through ATT. Findings involving PCA were less stable across measurement specifications. Excluding PI2 substantially increased the coefficient for the association between ATT and PI and the proportion of variance explained in PI, and a direct association between PBC and PI emerged only in specifications excluding PI2. These increases were not interpreted as evidence that the reduced specifications were superior, because removal of PI2 also increased the empirical similarity between the remaining PI indicators and ATT; furthermore, in the reduced-item sensitivity specification, PBC was represented by the single remaining item PBC2 (Supplementary Table S7).

## 4. Discussion

The primary adjusted model identified a coherent but qualified pattern of associations. HC and PBC were consistently positively associated with ATT, and both showed positive indirect statistical associations with PI through ATT across composite and latent-variable sensitivity analyses. PCA was positively associated with ATT in the primary adjusted model, but its direct and indirect findings were more sensitive to measurement specification. FI did not show a stable independent association with ATT or PI. The model explained 29.4% of ATT variance and 43.2% of PI variance, indicating that the selected psychosocial constructs and background covariates captured a meaningful portion of individual differences in purchase intention while leaving substantial variance unexplained. These results are best interpreted within a TPB-informed framework rather than as evidence that the study formally extended or validated the canonical TPB [9,14,15].

ATT showed the largest coefficient in relation to PI in the primary adjusted composite model, consistent with studies in which favorable evaluations were positively associated with intentions to purchase organic vegetables or other foods [21,22,23]. However, this finding also represents the principal measurement caution of the study. Although the focused two-factor analysis statistically distinguished ATT from PI, the high latent correlation (0.933), HTMT ratio (0.925), and failure of the Fornell–Larcker criterion indicated limited discriminant validity. The latent SEMs yielded substantially larger coefficients for the association between ATT and PI than the primary composite model, and exclusion of PI2 further increased both this coefficient and the proportion of variance explained in PI. Because PI2 captures intended effort within a near-future time frame and was less strongly correlated with the ATT indicators than PI1 and PI3, its removal appears to make the remaining PI score more empirically similar to ATT rather than demonstrably improving construct validity. Consequently, the estimated association between ATT and PI, as well as the indirect statistical associations that incorporate this coefficient, may be inflated by empirical overlap between the constructs.

HC and PBC were the most stable antecedent findings. Greater HC was associated with a more favorable ATT and showed a positive indirect statistical association with PI, whereas its direct association with PI was not supported after adjustment. This is consistent with previous studies linking health consciousness to favorable food-related attitudes and purchase intentions [16,17,18,19,20]. PBC, operationalized in the primary analysis as personal control and confidence, likewise showed a positive association with ATT and a robust positive indirect statistical association with PI. Previous TPB-based studies have reported positive associations between PBC and intention in the context of organic or healthy food purchasing [14,17,22,23]. However, the direct association between PBC and PI was not stable across the present analyses: it was not supported in the primary adjusted model or the hybrid latent SEM and emerged only in selected alternative measurement specifications. This inconsistency warrants caution because the two-item PBC composite showed borderline reliability (0.619), PBC1 had a comparatively weak loading in the original CFA, and the complete three-item version combined personal control/confidence with resource and time constraints.

FI was not independently associated with ATT or PI in the primary adjusted model, and its indirect statistical association through ATT was not supported. Unlike conventional TPB subjective norm, FI specifically captured approval, expectations, influence, and recommendations from a partner or family [30,31,32,33]. Normative effects have varied across dietary and food-purchasing studies [14,15,22,24,25,27,41], and differences in household or marital context may affect the relevance of family input [42]. Thus, the present findings indicate no stable independent association for this family-specific measure but do not imply that family processes are unimportant for household food decisions.

PCA captured perceived proximity of stores, perceived product variety, and perceived availability of fruits and vegetables at usual shopping locations. In the primary adjusted composite model it was positively associated with ATT and showed a small positive indirect statistical association through ATT, but neither the total association with PI nor the result across all measurement specifications was stable. This distinction matters because the construct represents perceived access, not objective retail availability. Prior research has linked perceptions of the food environment and availability with fruit and vegetable selection or intake [34,35,36,37,38,39,40], yet the present findings cannot establish that increasing the number of stores, product variety, or physical availability would change purchase intention.

The contribution of the present study lies primarily in jointly examining personal, family-related normative, control-related, and perceived-access correlates of fruit and vegetable purchase intention in a Peruvian convenience sample, rather than in proposing a new extension of the TPB. Contemporary syntheses describe food choice as the product of interacting individual, social, and contextual influences [43], and recent Latin American evidence similarly indicates that health consciousness, attitude, perceived control, and other decision-related factors are associated with healthy-product purchase intention [44]. Choice-experiment evidence further suggests that health-related information and consumer characteristics can shape evaluations and willingness to pay for fresh produce [45].

The adjusted PI model accounted for 43.2% of variance, compared with 39.6% in the unadjusted model. This difference should not be attributed to the focal psychosocial constructs alone because the adjusted model also included background sociodemographic covariates. More than half of PI variance remained unexplained, which is consistent with the broader view that food-related intentions may depend on additional factors such as habitual purchasing, affordability, perceived freshness and quality, sensory preferences, household decision processes, and situational constraints [39,40,42,43].

### 4.1. Practical Implications

The findings may inform formative research and the development of future strategies related to fruit and vegetable purchasing, although the cross-sectional design does not establish that modifying any of the measured constructs would change purchasing behavior. HC and perceived personal control/confidence were the most consistent antecedent correlates and may therefore be useful domains to assess when developing or tailoring educational strategies related to fruit and vegetable purchasing. Future strategies could explore ways of connecting purchasing decisions with health goals and strengthening consumers’ confidence in planning and carrying out fruit and vegetable purchases; the effectiveness of such approaches should be evaluated prospectively or experimentally. Perceived convenience and availability may also be relevant when tailoring strategies to local purchasing contexts by considering consumers’ perceptions of store proximity, product variety, and availability, preferably alongside objective food-environment information. Likewise, although FI did not show an independent association in the present model, family-level approaches may still warrant consideration in contexts involving shared household food purchasing. Overall, the findings identify domains that may be considered in formative assessment and future intervention research rather than established mechanisms of behavioral change.

### 4.2. Strengths, Limitations, and Future Research

Strengths include the joint assessment of personal, family-related normative, perceived-control, and perceived-access correlates; transparent reporting of the complete 21-item instrument; ordinal CFA; robust HC3 inference; bootstrap estimation of indirect and total statistical associations; explicit common-method diagnostics; and complementary sensitivity analyses using unadjusted composites, latent-variable SEMs, and alternative item specifications.

Several limitations are central to interpretation. First, the cross-sectional design does not establish temporal ordering or causality, so the indirect associations are statistical decompositions within the specified model rather than causal mediation. Second, the non-probability online sample recruited through WhatsApp and Facebook Messenger was predominantly female (73.8%), university educated (76.9%), and Lima based (66.2%); consequently, the findings characterize this convenience sample and should not be generalized to the Peruvian adult population. Third, all focal variables were collected by self-report in the same questionnaire. Although the single-factor CFA and Harman diagnostic did not support dominance by one general factor, common-method variance, social-desirability effects, and response-style effects cannot be excluded.

Measurement limitations were also important. HC2 and PBC3 were excluded from the primary composites after measurement and content assessment in the same sample used for structural analysis, and PI4 was excluded on content-validity grounds; these decisions require independent validation. HC and PBC were represented by two-item primary scores, with borderline reliability for PBC. Most importantly, ATT and PI showed limited discriminant validity. Although the focused two-factor model statistically distinguished the constructs, the high latent correlation (0.933), HTMT ratio (0.925), and failure of the Fornell–Larcker criterion indicated substantial empirical overlap. Accordingly, the estimated association between ATT and PI, as well as the indirect statistical associations incorporating this coefficient, may be inflated by construct overlap and should be interpreted cautiously.

Results involving PCA and some direct associations between PBC and PI were also sensitive to item specification. The outcome of this study was purchase intention by design; therefore, the results should be delimited to self-reported intention and not extrapolated to actual purchasing or dietary intake.

Future studies should prioritize probability-based and regionally diverse samples, independently validate the revised measurement specifications, and use longitudinal or temporally separated designs to clarify the ordering of ATT and PI. Measures of purchase intention should use a consistent future time frame and clearly distinguish willingness, planning, effort, continuation, and recommendation. Studies that extend this work to observed purchasing or dietary intake should treat those as distinct outcomes rather than assuming that intention translates directly into behavior. Objective indicators of retail access could also be paired with perceived convenience and availability, and measurement invariance across major demographic groups should be evaluated before substantive group comparisons.

## 5. Conclusions

In this non-probability convenience sample of adults residing in Peru—73.8% female, 76.9% university educated, and 66.2% residing in Lima—HC and PBC showed the most consistent antecedent associations with ATT and positive indirect statistical associations with PI through attitude. PCA showed less stable evidence, whereas FI did not show a stable independent association. ATT was strongly associated with PI, although the limited discriminant validity between these constructs warrants cautious interpretation. Because the sample was non-random and demographically imbalanced, these findings should not be generalized to the Peruvian adult population and pertain to self-reported purchase intention rather than actual purchasing or dietary intake.

## Figures and Tables

**Table 1:** Sociodemographic and background characteristics of the study participants (*n* = 458).

Characteristic	Category	*n*	% *
**Age group (years)**	18–30	78	17.0
	31–40	88	19.2
	41–50	141	30.8
	51–60	151	33.0
**Sex**	Male	120	26.2
	Female	338	73.8
**Educational level**	Primary	1	0.2
	Secondary	29	6.3
	University	352	76.9
	Technical	76	16.6
**Marital status**	Single	176	38.4
	Cohabiting	55	12.0
	Married	188	41.0
	Divorced	32	7.0
	Widowed	7	1.5
**Employment status**	Employee	300	65.5
	Employer	20	4.4
	Self-employed	123	26.9
	Pensioner	15	3.3
**Monthly household income (PEN)**	<1300	61	13.3
	1301–2480	90	19.7
	2481–3,970	128	27.9
	3971–7020	97	21.2
	7021–12,660	43	9.4
	>12,660	39	8.5
**Region of residence**	Lima	303	66.2
	Callao	12	2.6
	Other regions	143	31.2
**Physical activity level**	≥30 min/day moderate or ≥15 min/day vigorous	86	18.8
	<30 min/day moderate or <15 min/day vigorous	194	42.4
	No physical activity	178	38.9

Note. PEN = Peruvian sol. * Relative frequencies were calculated using the complete analytical sample (*n* = 458). For multivariable analyses, primary and secondary education were combined as “secondary or less”; married and cohabiting participants were combined as “with partner”, and divorced and widowed participants as “previously married/cohabiting”. All other covariate categories were retained as shown, except in the geographic sensitivity analysis, in which Lima and Callao were combined.

**Table 2:** Descriptive statistics and distributional properties of the primary study constructs.

Study Construct	M	SD	Median	IQR (Q1–Q3)	Min	Max
HC	4.05	0.79	4.00	4.00–4.50	1.00	5.00
FI	3.55	0.74	3.50	3.00–4.00	1.00	5.00
PBC	4.00	0.74	4.00	3.50–4.50	1.00	5.00
PCA	3.47	0.97	3.67	2.67–4.00	1.00	5.00
ATT	4.35	0.61	4.25	4.00–5.00	1.00	5.00
PI	4.20	0.61	4.00	4.00–4.67	1.00	5.00

Note. M = mean; SD = standard deviation; IQR = interquartile range; Min = minimum; Max = maximum; HC = health consciousness; FI = family influence; PBC = perceived behavioral control; PCA = perceived convenience and availability; ATT = attitude; PI = purchase intention. Primary composite scores ranged from 1 to 5, with higher scores indicating higher levels of each construct. HC was based on HC1 and HC3; FI on FI1–FI4; PBC on PBC1 and PBC2; PCA on the three reverse-scored PCA items; ATT on ATT1–ATT4; and PI on PI1–PI3.

**Table 3:** Correlations, reliability, and collinearity diagnostics of the primary study constructs.

Construct	1	2	3	4	5	6	Reliability	Tolerance	VIF
1. HC	—						0.731 ^a^	0.723	1.384
2. FI	0.206 ***	—					0.744 ^a^	0.884	1.131
3. PBC	0.311 ***	0.011	—				0.619 **^a^**	0.837	1.194
4. PCA	0.088	−0.046	−0.035	—			0.858 ^b^	0.862	1.160
5. ATT	0.502 ***	0.091	0.321 ***	0.182 ***	—		0.929 ^b^	0.706	1.417
6. PI	0.367 ***	0.132 **	0.222 ***	0.044	0.572 ***	—	0.799 ^b^	—	—

Note. Values below the diagonal are Spearman rank correlations ($r_{s}$). HC = health consciousness; FI = family influence; PBC = perceived behavioral control; PCA = perceived convenience and availability; ATT = attitude; PI = purchase intention. ^a^ Reliability was estimated using the ordinal Spearman–Brown coefficient based on the polychoric correlation between the two items. ^b^ Reliability was estimated using ordinal McDonald’s total omega ($\omega_{t}$) based on polychoric correlations. Tolerance and variance inflation factor (VIF) values correspond to the focal predictors in the fully sociodemographically adjusted purchase-intention model. PI is the dependent variable in that model and therefore has no corresponding tolerance or VIF. ** *p* < 0.01; *** *p* < 0.001.

**Table 4:** Adjusted direct, indirect, and total statistical associations with fruit and vegetable purchase intention.

**Panel A. Direct associations**					
**Association**	**β**	**Robust SE**	**t**	***p***	**95% CI**
HC → ATT	0.331	0.068	4.861	<0.001	0.197–0.465
FI → ATT	0.051	0.053	0.958	0.339	−0.054–0.155
PBC → ATT	0.228	0.055	4.134	<0.001	0.120–0.337
PCA → ATT	0.096	0.042	2.306	0.022	0.014–0.178
ATT → PI	0.568	0.054	10.590	<0.001	0.463–0.674
HC → PI	0.062	0.056	1.116	0.265	−0.047–0.171
FI → PI	0.055	0.044	1.256	0.210	−0.031–0.142
PBC → PI	0.037	0.046	0.792	0.429	−0.054–0.128
PCA → PI	−0.081	0.042	−1.937	0.053	−0.162–0.001
**Panel B. Indirect statistical associations through ATT**					
**Association**	**β**	**Bootstrap 95% CI**			
HC → ATT → PI	0.188	0.117–0.265			
FI → ATT → PI	0.029	−0.025–0.082			
PBC → ATT → PI	0.130	0.073–0.190			
PCA → ATT → PI	0.055	0.010–0.104			
**Panel C. Total statistical associations with PI**					
**Association**	**β**	**Bootstrap 95% CI**			
HC total → PI	0.250	0.141–0.361			
FI total → PI	0.084	−0.020–0.186			
PBC total → PI	0.167	0.056–0.266			
PCA total → PI	−0.026	−0.119–0.065			

Note. HC = health consciousness; FI = family influence; PBC = perceived behavioral control; PCA = perceived convenience and availability; ATT = attitude; PI = purchase intention. β denotes standardized regression coefficients. Models were adjusted for sex, age group, educational level, marital status, household-income category, and region of residence. HC3 robust standard errors and confidence intervals were used for direct associations. Confidence intervals for indirect and total statistical associations were obtained from 5000 nonparametric bootstrap resamples, with construct scores re-standardized within each resample. *R*^2^ = 0.294 for ATT and *R*^2^ = 0.432 for PI.

## Data Availability

The anonymized data supporting the findings of this study are available from the corresponding author upon reasonable request. Public sharing of the dataset is restricted due to ethical and participant privacy considerations, in accordance with the conditions established by the approving ethics committee.

## Supplementary Materials

The following supporting information can be downloaded at: https://www.mdpi.com/article/10.3390/nu18183033/s1, Table S1: Questionnaire items, English translations, coding, analytical status, and sources. Table S2: Global fit and admissibility of the confirmatory measurement models. Table S3: Standardized factor loadings, reliability, and convergent validity. Table S4: Latent correlations, HTMT ratios, Fornell–Larcker assessment, and focused attitude–purchase intention discriminant-validity analysis. Table S5: Unadjusted direct, indirect, and total statistical associations in the primary composite path model. Table S6: Latent-variable structural equation model sensitivity analyses. Table S7: Sensitivity of the adjusted composite path model to alternative item specifications. Table S8: Common-method variance diagnostics.
