# Supplementary material for: Personal, Normative, and Access-Related Correlates of Fruit and Vegetable Purchase Intention in Peruvian Adults
Source: Nutrients. 2026 Sep 16;18(18):3033. doi: 10.3390/nu18183033 (PMC13610518; doi:10.3390/nu18183033)
Supplement: Supplementary file 1 [file nutrients-18-03033-s001.zip › nutrients-4510968-supplementary.pdf]

Supplementary Table S1. Questionnaire items, English translations, coding, analytical status, and sources

Construct	Code	Item administered in Spanish	English translation	Coding and analytical status	Source
Health consciousness	HC1	Elijo frutas y verduras con cuidado para garantizar una buena salud.	I carefully choose fruits and vegetables to ensure good health.	Directly scored; retained in primary composite	Yadav and Pathak, 2016
Health consciousness	HC2	No me considero un consumidor preocupado por su salud.	I do not consider myself a health-conscious consumer.	Reverse-coded; excluded from primary composite after measurement assessment; retained in original-item sensitivity analyses	Yadav and Pathak, 2016
Health consciousness	HC3	Pienso a menudo en temas relacionados con la salud.	I often think about health-related issues.	Directly scored; retained in primary composite	Yadav and Pathak, 2016
Family influence	FI1	La aprobación de mi pareja o familia es importante para mí a la hora de comprar frutas y verduras.	The approval of my partner or family is important to me when purchasing fruits and vegetables.	Directly scored; retained in primary composite	Chauke and Duh, 2019
Family influence	FI2	Mi familia espera que elija frutas y verduras.	My family expects me to choose fruits and vegetables.	Directly scored; retained in primary composite	Chauke and Duh, 2019
Family influence	FI3	Mi familia me influye para que compre frutas y verduras.	My family influences me to purchase fruits and vegetables.	Directly scored; retained in primary composite	Chauke and Duh, 2019
Family influence	FI4	Mi familia me recomienda que consuma frutas y verduras.	My family recommends that I consume fruits and vegetables.	Directly scored; retained in primary composite	Chauke and Duh, 2019
Perceived behavioral control	PBC1	Comprar o no comprar frutas y verduras depende totalmente de mí.	Whether or not I purchase fruits and vegetables depends entirely on me.	Directly scored; retained in primary composite	Yadav and Pathak, 2016
Perceived behavioral control	PBC2	Estoy seguro de que, si quiero, puedo comprar frutas y verduras.	I am confident that, if I want to, I can purchase fruits and vegetables.	Directly scored; retained in primary composite	Yadav and Pathak, 2016
Perceived behavioral control	PBC3	No tengo recursos ni tiempo para comprar frutas y verduras.	I do not have the resources or time to purchase fruits and vegetables.	Reverse-coded; excluded from primary composite because it reflects external resource/time constraints and showed poor coherence with the personal-control indicators; retained in original-item sensitivity analyses	Yadav and Pathak, 2016
Perceived convenience and availability	PCA1	Hay pocas tiendas que venden frutas y verduras cerca de donde vivo o trabajo.	There are few stores selling fruits and vegetables near where I live or work.	Reverse-coded; retained in primary composite	Kamboj et al., 2023

Perceived convenience and availability	PCA2	Las tiendas donde compro con frecuencia no venden una variedad de frutas y verduras.	The stores where I frequently shop do not sell a variety of fruits and vegetables.	Reverse-coded; retained in primary composite	Kamboj et al., 2023
Perceived convenience and availability	PCA3	Las frutas y verduras solo están disponibles en algunas tiendas o mercados.	Fruits and vegetables are available only in certain stores or markets.	Reverse-coded; retained in primary composite	Kamboj et al., 2023
Attitude	ATT1	Me gusta la idea de comprar frutas y verduras.	I like the idea of purchasing fruits and vegetables.	Directly scored; retained in primary composite	Eberle et al., 2022
Attitude	ATT2	Comprar frutas y verduras es una buena elección.	Purchasing fruits and vegetables is a good choice.	Directly scored; retained in primary composite	Eberle et al., 2022
Attitude	ATT3	Tengo una actitud favorable hacia la compra de frutas y verduras.	I have a favorable attitude toward purchasing fruits and vegetables.	Directly scored; retained in primary composite	Eberle et al., 2022
Attitude	ATT4	Comprar frutas y verduras sería agradable.	Purchasing fruits and vegetables would be pleasant.	Directly scored; retained in primary composite	Eberle et al., 2022
Purchase intention	PI1	Estoy dispuesto a comprar frutas y verduras mientras hago mis compras.	I am willing to purchase fruits and vegetables while doing my shopping.	Directly scored; retained in primary composite	Kamboj et al., 2023
Purchase intention	PI2	Haré un esfuerzo para comprar frutas y verduras en un futuro próximo.	I will make an effort to purchase fruits and vegetables in the near future.	Directly scored; retained in primary composite despite its comparatively lower loading because it captures intended effort/near-future intention; exclusion was examined in sensitivity analyses	Kamboj et al., 2023
Purchase intention	PI3	Seguiré comprando frutas y verduras.	I will continue purchasing fruits and vegetables.	Directly scored; retained in primary composite	Kamboj et al., 2023
Purchase intention	PI4	Recomendaría comprar frutas y verduras a un amigo o familiar.	I would recommend purchasing fruits and vegetables to a friend or family member.	Directly scored; excluded from primary composite on content-validity grounds because it assesses recommendation to others rather than the respondent's own purchase intention; retained in sensitivity analyses	Kamboj et al., 2023

Note. All items were rated on a five-point Likert-type scale ranging from 1 (*strongly disagree*) to 5 (*strongly agree*). Negatively worded items (HC2, PBC3, PCA1–PCA3) were reverse-coded as 6 minus the original response. Primary composite scores were calculated as the mean of the retained items: HC = HC1 and HC3; FI = FI1–FI4; PBC = PBC1 and PBC2; PCA = reverse-coded PCA1–PCA3; ATT = ATT1–ATT4; and PI = PI1–PI3. Higher scores represented higher levels of each construct. Items excluded from the primary composites were retained in prespecified sensitivity analyses. The complete original 21-item specification was also evaluated as a sensitivity analysis.

Supplementary Table S2. Global fit and admissibility of the confirmatory measurement models

Measurement model	Observed indicators	χ^2scaled (df)	CFIscaled	TLIscaled	RMSEA scaled (95% CI)	SRMR	Converged	Admissible solution
**Original six-factor CFA**	21	952.121 (174)***	0.940	0.928	0.099 (0.093–0.105)	0.083	Yes	Yes
**Hybrid reliability-corrected six-factor measurement model**	16	484.356 (91)***	0.956	0.942	0.097 (0.089–0.106)	0.073	Yes	Yes

Note. ***$p < 0.001$ for the model χ^2 test. CFA = confirmatory factor analysis; CFI = comparative fit index; TLI = Tucker–Lewis index; RMSEA = root mean square error of approximation; SRMR = standardized root mean square residual. The original CFA included all 21 administered ordinal items and specified six correlated factors: health consciousness (HC), family influence (FI), perceived behavioral control (PBC), perceived convenience and availability (PCA), attitude (ATT), and purchase intention (PI). It was estimated using WLSMV for ordinal indicators with delta parameterization and standardized latent-variable scaling. The hybrid measurement model was used as a measurement-error sensitivity analysis because the primary HC and PBC composites contained only two indicators. HC and PBC were represented by standardized composite scores with residual error fixed according to their ordinal Spearman–Brown reliability estimates, whereas FI, PCA, ATT, and PI were represented by their item-level indicators; PI comprised PI1–PI3. The two models differ in indicators and parameterization and therefore should not be interpreted as nested model comparisons. Reported CFI, TLI, and RMSEA values are the scaled WLSMV indices.

Supplementary Table S3. Standardized factor loadings, reliability, and convergent validity

Construct	Item	Standardized loading	CR[a]	AVE[a]	Primary-score reliability[b]	Primary analytical status
**Health consciousness (HC)**	HC1	0.683	0.655	0.403	0.731[c]	Retained
	HC2_R	0.398				Excluded from primary composite
	HC3	0.764				Retained
**Family influence (FI)**	FI1	0.475	0.744	0.428	0.744[d]	Retained
	FI2	0.788				Retained
	FI3	0.661				Retained
	FI4	0.654				Retained
**Perceived behavioral control (PBC)**	PBC1	0.359	0.600	0.356	0.619[c]	Retained
	PBC2	0.808				Retained
	PBC3_R	0.536				Excluded from primary composite
**Perceived convenience and availability (PCA)**	PCA1_R	0.823	0.859	0.669	0.858[d]	Retained
	PCA2_R	0.836				Retained
	PCA3_R	0.795				Retained
**Attitude (ATT)**	ATT1	0.890	0.930	0.770	0.929[d]	Retained
	ATT2	0.904				Retained
	ATT3	0.854				Retained
	ATT4	0.861				Retained
**Purchase intention (PI)**	PI1	0.887	0.873	0.651	0.799[d]	Retained
	PI2	0.381				Retained
	PI3	0.912				Retained
	PI4	0.915				Excluded from primary composite

Note. Standardized loadings, composite reliability (CR), and average variance extracted (AVE) correspond to the original 21-item six-factor WLSMV confirmatory factor model. Reverse-scored indicators are denoted by the suffix _R. [a] CR and AVE were calculated from standardized CFA loadings and therefore refer to the original item-level measurement specification. [b] Primary-score reliability refers to the composite scores used in the primary path analysis and should not be interpreted as belonging to the original 21-item CFA specification. [c] Ordinal Spearman–Brown coefficient based on the polychoric correlation between the two retained items. [d] Ordinal McDonald's total omega (ω_t) based on polychoric correlations. HC2 and PBC3 were excluded from the primary composites following measurement and content assessment; PI4 was excluded on content-validity grounds because it assessed recommendation to others rather than the respondent's own purchase intention. PI2 was retained in the primary composite despite its comparatively low loading because it captures intended effort/near-future intention and its exclusion was examined separately in sensitivity analyses.

Supplementary Table S4. Latent correlations, HTMT ratios, Fornell–Larcker assessment, and focused attitude–purchase intention discriminant-validity analysis

Panel A. Latent correlations, HTMT ratios, and square roots of AVE

Construct	HC	FI	PBC	PCA	ATT	PI
**HC**	0.634	0.431	0.723	0.282	0.745	0.666
**FI**	0.387	0.654	0.419	0.087	0.234	0.291
**PBC**	0.612	0.190	0.597	0.328	0.775	0.753
**PCA**	0.234	−0.033	0.228	0.818	0.213	0.141
**ATT**	0.702	0.240	0.710	0.212	0.877	0.925
**PI**	0.634	0.273	0.730	0.121	0.933	0.807

Note. Values below the diagonal are latent-factor correlations estimated from the original 21-item six-factor WLSMV confirmatory factor model. Values above the diagonal are heterotrait–monotrait ratios (HTMT) based on polychoric item correlations. Bold diagonal values are the square roots of average variance extracted (AVE). HC = health consciousness; FI = family influence; PBC = perceived behavioral control; PCA = perceived convenience and availability; ATT = attitude; PI = purchase intention.

Panel B. Focused discriminant-validity analysis of ATT and PI

Model	χ^2scaled	df	CFIscaled	TLIscaled	RMSEA scaled	SRMR	Interpretation
**Two-factor ATT–PI model**	108.101	19	0.989	0.984	0.101	0.030	Statistically preferred
**One-factor ATT–PI model**	182.425	20	0.981	0.973	0.133	0.038	Inferior fit
**Robust difference test**	$\Delta\chi^2$ = 63.009	Δdf = 1	—	—	—	—	$p < 0.001$

Note. The two-factor model specified ATT and PI as correlated but distinct latent variables, whereas the one-factor model specified all ATT and PI indicators as loading on a single common factor. The robust WLSMV chi-square difference test favored the two-factor specification, $\Delta\chi^2(1) = 63.009$, $p < 0.001$. Nevertheless, the very high latent correlation (0.933), HTMT ratio (0.925), and failure of the Fornell–Larcker criterion indicate limited discriminant validity between ATT and PI. Thus, the constructs were statistically distinguishable but showed substantial empirical overlap.

Supplementary Table S5. Unadjusted direct, indirect, and total statistical associations in the primary composite path model

Panel A. Direct associations

Association	β	Robust SE	t	p	95% CI
HC → ATT	0.337	0.070	4.847	<0.001	0.201–0.474
FI → ATT	0.061	0.053	1.144	0.253	−0.044–0.166
PBC → ATT	0.252	0.058	4.344	<0.001	0.138–0.366
PCA → ATT	0.102	0.037	2.744	0.006	0.029–0.175
ATT → PI	0.575	0.053	10.768	<0.001	0.470–0.680
HC → PI	0.040	0.054	0.755	0.451	−0.065–0.146
FI → PI	0.072	0.043	1.687	0.092	−0.012–0.157
PBC → PI	0.062	0.045	1.367	0.172	−0.027–0.151
PCA → PI	−0.062	0.041	−1.532	0.126	−0.142–0.018

Panel B. Indirect statistical associations through ATT

Association	β	Bootstrap 95% CI
HC → ATT → PI	0.194	0.121–0.274
FI → ATT → PI	0.035	−0.020–0.094
PBC → ATT → PI	0.145	0.083–0.210
PCA → ATT → PI	0.059	0.017–0.104

Panel C. Total statistical associations with PI

Association	β	Bootstrap 95% CI
HC total → PI	0.234	0.127–0.346
FI total → PI	0.108	0.003–0.211
PBC total → PI	0.207	0.096–0.308
PCA total → PI	−0.004	−0.090–0.087

Note. β denotes standardized regression coefficients. HC3 robust standard errors and confidence intervals were used for direct associations. Confidence intervals for indirect and total statistical associations were obtained from 5000 nonparametric bootstrap resamples, with construct scores re-standardized within each resample. No sociodemographic covariates were included. $R^2 = 0.247$for ATT and $R^2 = 0.396$for PI. HC = health consciousness; FI = family influence; PBC = perceived behavioral control; PCA = perceived convenience and availability; ATT = attitude; PI = purchase intention.

Supplementary Table S6. Latent-variable structural equation model sensitivity analyses

Panel A. Global fit and technical diagnostics

**Model**	**χ^2scaled (df)**	**CFIscaled**	**TLIscaled**	**RMSEA scaled (95% CI)**	**SRMR**	**R^2 ATT**	**R^2 PI**
**Full 21-item latent SEM**	952.121 (174)	0.940	0.928	0.099 (0.093–0.105)	0.083	0.619	0.894
**Hybrid reliability-corrected latent SEM**	484.356 (91)	0.956	0.942	0.097 (0.089–0.106)	0.073	0.396	0.881

Panel B. Standardized direct structural associations

**Association**	**Full 21-item SEM β**	**p**	**Hybrid SEM β**	**p**
HC → ATT	0.433	<0.001	0.361	<0.001
FI → ATT	−0.012	0.803	0.057	0.199
PBC → ATT	0.445	<0.001	0.332	<0.001
PCA → ATT	0.008	0.832	0.186	<0.001
ATT → PI	0.881	<0.001	0.931	<0.001
HC → PI	−0.093	0.165	−0.053	0.117
FI → PI	0.063	0.131	0.059	0.171
PBC → PI	0.169	0.003	0.066	0.185
PCA → PI	−0.080	0.033	−0.074	0.077

Panel C. Indirect statistical associations through ATT

**Association**	**Full 21-item SEM β**	**Inference**	**Hybrid SEM β**	**Inference**
HC → ATT → PI	**0.381**	$p < 0.001$	0.336	$p < 0.001$
FI → ATT → PI	−0.010	Not supported	0.053	Not supported
PBC → ATT → PI	0.392	$p < 0.001$	0.310	$p < 0.001$
PCA → ATT → PI	0.007	Not supported	0.173	$p < 0.001$

Panel D. Total standardized associations with PI

**Association**	**Full 21-item SEM β**	**Hybrid SEM β**
HC total → PI	**0.288**	**0.283**
FI total → PI	0.052	0.112
PBC total → PI	0.561	0.375
PCA total → PI	−0.073	0.098

Note. SEM = structural equation model; HC = health consciousness; FI = family influence; PBC = perceived behavioral control; PCA = perceived convenience and availability; ATT = attitude; PI = purchase intention. Both models were estimated using WLSMV. The full 21-item SEM represented all six constructs as item-level latent variables using the complete administered item set. In the hybrid model, HC and PBC were represented by standardized composite indicators with residual measurement error fixed according to their ordinal Spearman–Brown reliability estimates, whereas FI, PCA, ATT, and PI were modeled using item-level indicators; PI included PI1–PI3. Standardized coefficients (β) are reported. The two models differ in measurement specification and therefore should be interpreted as complementary sensitivity analyses rather than directly competing models. Global fit indices are identical to those of the corresponding measurement models because the structural relations among the latent variables essentially saturate the construct-level covariance structure; consequently, global fit should not be interpreted as validation of individual theoretical paths.

Supplementary Table S7. Sensitivity of the adjusted composite path model to alternative item specifications

Panel A. Direct structural associations

**Association**	**A. Primary specification**	**B. Original 21-item specification**	**C. PI4 retained**	**D. PI2 excluded**	**E. Weak-indicator exclusion**
HC → ATT	0.331***	0.317***	0.331***	0.331***	0.282***
FI → ATT	0.051	0.093	0.051	0.051	0.087
PBC → ATT	0.228***	0.276***	0.228***	0.228***	0.308***
PCA → ATT	0.096*	0.039	0.096*	0.096*	0.074
ATT → PI	0.568***	0.652***	0.643***	0.705***	0.672***
HC → PI	0.062	0.008	0.047	0.036	0.014
FI → PI	0.055	0.062	0.051	0.008	0.059
PBC → PI	0.037	0.055	0.044	0.101*	0.142**
PCA → PI	−0.081	−0.076*	−0.071	−0.041	−0.047

Panel B. Indirect statistical associations through ATT

**Association**	**A. Primary specification**	**B. Original 21-item specification**	**C. PI4 retained**	**D. PI2 excluded**	**E. Weak-indicator exclusion**
HC → ATT → PI	0.188 [0.117, 0.265]	0.207 [0.134, 0.284]	0.213 [0.134, 0.298]	0.234 [0.147, 0.326]	0.190 [0.108, 0.279]
FI → ATT → PI	0.029 [−0.025, 0.082]	0.061 [−0.003, 0.124]	0.033 [−0.029, 0.093]	0.036 [−0.031, 0.102]	0.059 [−0.005, 0.120]
PBC → ATT → PI	0.130 [0.073, 0.190]	0.180 [0.114, 0.249]	0.147 [0.083, 0.211]	0.161 [0.093, 0.230]	**0.207 [0.133, 0.286]**
PCA → ATT → PI	0.055 [0.010, 0.104]	0.025 [−0.027, 0.078]	0.062 [0.011, 0.118]	0.068 [0.012, 0.130]	0.050 [−0.002, 0.108]

Panel C. Explained variance

**Outcome**	**A. Primary specification**	**B. Original 21-item specification**	**C. PI4 retained**	**D. PI2 excluded**	**E. Weak-indicator exclusion**
R^2 ATT	0.294	0.314	0.294	0.294	0.340
R^2 PI	0.432	0.509	0.510	0.604	0.615

Note. HC = health consciousness; FI = family influence; PBC = perceived behavioral control; PCA = perceived convenience and availability; ATT = attitude; PI = purchase intention. All models were adjusted for sex, age group, educational level, marital status, household-income category, and region of residence. Direct-path inference used HC3 robust standard errors. Indirect statistical associations and their 95% confidence intervals were estimated using 5000 nonparametric bootstrap resamples, with construct scores re-standardized within each resample. Specification A was the primary analysis (HC1/HC3; FI1–FI4; PBC1/PBC2; reverse-scored PCA1–PCA3; ATT1–ATT4; PI1–PI3). Specification B used all 21 originally administered items. Specification C retained PI4 in addition to PI1–PI3. Specification D excluded PI2, resulting in PI being represented by PI1 and PI3. Specification E excluded FI1, FI3, PBC1, and PI2 as a reviewer-requested sensitivity analysis; consequently, FI was represented by FI2 and FI4, PBC by the single remaining indicator PBC2, and PI by PI1 and PI3. Specification E should therefore be interpreted only as a sensitivity analysis and not as an alternative validated measurement model. *$p < 0.05$; ** $p < 0.01$; ***$p < 0.001$.

Supplementary Table S8. Common-method variance diagnostics

Panel A. Comparison of the hypothesized six-factor model with a single-factor CFA

Model	χ^2scaled	df	CFIscaled	TLIscaled	RMSEA scaled (95% CI)	SRMR
**Six-factor CFA**	952.121	174	0.940	0.928	0.099 (0.093–0.105)	0.083
**Single-factor CFA**	3188.786	189	0.769	0.743	0.186 (0.181–0.192)	0.136

Robust scaled chi-square difference: $\Delta\chi^2(15) = 1195.3$, $p < 0.001$.

Panel B. Harman single-factor diagnostic based on polychoric correlations

Diagnostic	Result
First unrotated factor, SS loadings	6.943
Variance accounted for by first factor	33.1%

Note. CFA = confirmatory factor analysis; CFI = comparative fit index; TLI = Tucker–Lewis index; RMSEA = root mean square error of approximation; SRMR = standardized root mean square residual. The six-factor CFA and single-factor CFA were estimated using WLSMV for the 21 ordinal questionnaire items. The robust scaled chi-square difference test compared the nested one- and six-factor specifications. As an additional descriptive diagnostic, Harman's single-factor analysis was conducted on the polychoric correlation matrix using an unrotated one-factor minimum-residual solution. The first factor accounted for 33.1% of the total variance. These analyses indicate that a single general factor does not adequately reproduce the covariance structure of the questionnaire; however, they do not demonstrate the absence of common-method variance.
